# Rare Pathogenic Variants in Genes Implicated in Glutamatergic Neurotransmission Pathway Segregate with Schizophrenia in Pakistani Families

**DOI:** 10.3390/genes12121899

**Published:** 2021-11-26

**Authors:** Ambrin Fatima, Uzma Abdullah, Muhammad Farooq, Yuan Mang, Mana M. Mehrjouy, Maria Asif, Zafar Ali, Niels Tommerup, Shahid M. Baig

**Affiliations:** 1 National Institute for Biotechnology and Genetic Engineering (NIBGE), Faisalabad 38000, Pakistan; ambrin.fatima@aku.edu (A.F.); uzma.abdullah@uaar.edu.pk (U.A.); masif@uni-koeln.de (M.A.); 2 Department of Cellular and Molecular Medicine, University of Copenhagen, 2200 Copenhagen, Denmark; mangyuan@gmail.com (Y.M.); mana@sund.ku.dk (M.M.M.); zafar@uswat.edu.pk (Z.A.); ntommerup@sund.ku.dk (N.T.); 3 Department of Biological and Biomedical Sciences, The Aga Khan University, Karachi 74800, Pakistan; 4 University Institute of Biochemistry and Biotechnology (UIBB), PMAS-Arid Agriculture University Rawalpindi, Rawalpindi 46000, Pakistan; 5 Department of Biotechnology, Institute of Biochemistry, Biotechnology and Bioinformatics (IBBB), The Islamia University of Bahawalpur, Bahawalpur 63100, Pakistan; 6 Department of Bioinformatics, Institute of Biochemistry, Biotechnology and Bioinformatics (IBBB), The Islamia University of Bahawalpur, Bahawalpur 63100, Pakistan; 7 Cologne Center for Genomics (CCG), University of Cologne, 50931 Cologne, Germany; 8 Centre for Biotechnology and Microbiology, University of Swat, Mingora 19130, Pakistan

**Keywords:** familial schizophrenia, duplication, rare variants, glutamatergic neurotransmission

## Abstract

Schizophrenia is a disabling neuropsychiatric disorder of adulthood onset with high heritability. Worldwide collaborations have identified an association of ~270 common loci, with small individual effects and hence weak clinical implications. The recent technological feasibility of exome sequencing enables the identification of rare variants of high penetrance that refine previous findings and improve risk assessment and prognosis. We recruited two multiplex Pakistani families, having 11 patients and 19 unaffected individuals in three generations. We performed genome-wide SNP genotyping, next-generation mate pairing and whole-exome sequencing of selected members to unveil genetic components. Candidate variants were screened in unrelated cohorts of 508 cases, 300 controls and fifteen families (with 51 affected and 47 unaffected individuals) of Pakistani origin. The structural impact of substituted residues was assessed through in silico modeling using iTASSER. In one family, we identified a rare novel microduplication (5q14.1_q14.2) encompassing critical genes involved in glutamate signaling, such as *CMYA5*, *HOMER* and *RasGRF2*. The second family segregates two ultra-rare, predicted pathogenic variants in the *GRIN2A* (NM_001134407.3: c.3505C>T, (p.R1169W) and in the *NRG3* NM_001010848.4: c.1951G>A, (p.E651K). These genes encode for parts of AMPA and NMDA receptors of glutamatergic neurotransmission, respectively, and the variants are predicted to compromise protein function by destabilizing their structures. The variants were absent in the aforementioned cohorts. Our findings suggest that rare, highly penetrant variants of genes involved in glutamatergic neurotransmission are contributing to the etiology of schizophrenia in these families. It also highlights that genetic investigations of multiplex, multigenerational families could be a powerful approach to identify rare genetic variants involved in complex disorders.

## 1. Introduction

Schizophrenia (SCZ) is a chronic neuropsychiatric disease afflicting around 1.1% of the population worldwide [1]. It manifests in late adolescence or early adulthood and present as a combination of multiple psychotic symptoms, such as hallucinations, delusions and abnormal moods and behavior. Most of the patients show disorganized thoughts and speech, accompanied by other cognitive deficits [2]. SCZ is believed to be a complex disorder caused by the interplay of a number of genetic and environmental factors [3]. Thus inherently, the underlying pathophysiological mechanism remains challenging to untangle. Family and twin studies have attributed strong genetic predisposition, estimating its heritability to be ~80% [4,5]. Its pathophysiology has been formally attributed to the cumulative effect of numerous common variants with modest individual effects, identified through global Genome-Wide Association Studies (GWAS) of thousands of individuals, the idea named as Common Disease Common Variants (CDCV) hypothesis. An aggregate score that is the sum of the weighted effect of all the markers together, the so-called, Polygenic Risk Score (PRS) gives a strong predictive ability for risk assessment in small target samples [6]. This later evidence emerged for the involvement of the few rare variants with moderate to high penetrance (Common Disease Rare Variant (CDRV) that contribute interactively [7,8], and akin to the near mendelian or oligogenic inheritance of the disease.

During the last decades of traditional genetics, efforts to identify genetic underpinnings of SCZ have largely been ineffective, mainly due to underpowered study designs. However, recent technical advancement in genomic analyses has opened new avenues of discovery by recruiting sporadic and familial cases at a large scale. One of the landmark cases in 2014 revealed 108 different loci associated with SCZ [9], which were soon updated to 145 [10], and recently to 270 [11]. In addition to these common variants, several rare variants have also been convincingly linked to SCZ, with the prominent contribution of large Copy Number Variations (CNVs). In general terms, larger CNVs (in the range of megabases) are associated with higher penetrance and a more severe phenotype, likely due to the involvement of multiple genes/regulatory regions [12,13,14,15,16,17,18]. It has implicated the disruption of diverse mechanisms, such as Glutamatergic, GABAergic and dopaminergic signaling, neuronal plasticity, in the etiology of SCZ, few of which could provide therapeutic targets.

Recently, a new paradigm has blossomed quickly; that is, the use of exome/genome sequencing to identify rare and ultra-rare highly penetrant variants contributing to the genetic etiology of SCZ [19,20,21,22,23,24,25,26]. Most of the SCZ-associated mutations and genes have been reported in sporadic cases through case-control or via patient-parents trio analyses [22,23,27,28]. Large multiplex families segregating SCZ hold great potential of discovery for relatively rare variants of high penetrance but have been rarely studied. Pakistan has reported a high incidence (1.5%) of schizophrenia [29], reaching up to 2.5% in rural areas of Punjab and Sindh Provinces [30]. The added burden is attributed to increased consanguinity and low disease awareness. Pakistani, being a big population, marked by high consanguinity and large family size, offers high potential to study the missing heritability of schizophrenia. Here we present families with multiple affected individuals that showed rare pathogenic variants co-segregating with phenotype.

## 2. Materials and Methods

### 2.1. Subjects

Two large multiplex SCZ families (A and B) of Pakistani origin were recruited in this study. The patients were diagnosed on the basis of clinical history and symptoms based on the Diagnostic and Statistical Manual of Mental Disorders, 4th Edition (DSM-IV). All the patients were diagnosed and confirmed by at least two senior psychiatrists of the Punjab Institute of Mental Health (PIMH) Lahore, Pakistan. Personal and familial history of psychiatric illness, tge onset of disease, past and present symptoms, lifestyle, previous admission(s) in psychiatric hospitals/clinics and history of pharmacological treatment were obtained through clinical records and by interviewing the patients and their immediate family members/caregivers. All participants/guardians signed written informed consent. Both affected and phenotypically healthy members of the families were interviewed to confirm their ailment. A comprehensive description of all affected individuals in both families is mentioned in Supplementary Table S1.

This study also recruited 15 additional families of ≥2 SCZ affected individuals and 508 unrelated SCZ patients (382 men and 126 women, with a mean age of 46 years) and 300 unrelated ethnicity-matched healthy controls (175 men and 125 women, with a mean age of 44 years) of Pakistani origin. The inclusion criterion for the control samples was over the age of 35 years and the absence of SCZ or any other psychotic illness in participants and their families.

### 2.2. Analysis for Copy Number Variations (CNVs)

Genomic DNA was isolated from peripheral blood samples using the organic method. To identify copy number variations, index patients (III:3 form family A and III:3, III:4, III:5, III:6, III:7, III:10, IV:1, IV:3, IV:4, IV:7, IV:8, IV:9, V:1, and V:4 form family B were genotyped with the Affymetrix Human Genome-Wide SNP array 6.0 following the manufacturer’s standard protocols. CNV screening was performed by the multi-algorithm approach using the Affymetrix Genotyping Console and Chromosomal Analysis Suite Software. Variants greater than 1 kb in size and involving 10 or more probes were classified as CNVs.

#### 2.2.1. Validation of CNV by Quantitative Real-Time PCR (qPCR)

The candidate duplication identified in family A was validated and analyzed for co-segregation with the disease in the family by TaqMan^®^ Copy number assays for the *RASGRF2*, *HOMER1* and *CMYA5* genes (Hs00311580-cn, Hs01799974-cn and Hs02033745-cn, respectively) (Applied Biosystems, Foster City, CA, USA). For relative quantification of copy number states, the *RNase P* TaqMan^®^ Copy Number reference assay was used. Each of the 10 µL duplex qPCR reaction mixtures contained 0.5 µL of both 20X Probes (target and reference assay), 10 ng genomic DNA and 5 µL of 2X TaqMan^®^ genotyping master mix. The reaction was performed on a 7500 Fast Real-Time PCR platform (Applied Biosystems, Bedford, MA, USA). Each sample was performed in triplicate. The data were collected and analyzed using the CopyCaller^®^ Software v2.0 (Applied Biosystems, Waltham, MA, USA).

#### 2.2.2. Next-Generation Mate Pair Sequencing (MPS)

To identify genomic organization of the duplication and check for complex chromosomal rearrangements, MPS using 1 μg of DNA was performed. Mate-pair libraries were prepared, sequenced and analyzed, as previously described [31], with modifications. Sequencing was performed on a HiSeq 2000 platform (Illumina) for 2 × 100 cycles, and the data were aligned to GRCh37/hg19.

#### 2.2.3. Validation of Breakpoints (BPs) with Sanger Sequencing

Genomic sequences flanking the BPs were extracted from the UCSC Genome Browser (GRCh37/hg19), and unique primers (sequences given in Supplementary Table S2) were designed to amplify the BP spanning regions from genomic DNA of all family members. The amplicons were analyzed on 1% agarose gel and sequenced in an ABI 3130xl genetic analyzer using a BigDye Terminator v. 3.1 Cycle Sequencing Kit (Applied Biosystems, Foster City, CA, USA) according to the manufacturer’s instructions. The sequences were aligned using Chromaspro (Technelysium Pty Ltd., South Brisbane, QLD, Australia) to distinguish the BPs.

### 2.3. Whole-Exome Sequencing (WES)

To identify potentially pathogenic variants causing SCZ, we subjected the three most distantly related SCZ patients III:4, IV:8 and one unaffected V:1 from family B to WES. The DNA of the selected individuals was briefly sheared, and libraries were prepared (Nextera Rapid Capture Expanded Exome Enrichment Kit) following the manufacturer’s protocol. The enriched, targeted, amplified products were subjected to 2 × 100 bp paired-end sequencing on an Illumina Hi-seq 2000 (Illumina, San Diego, CA, USA).

To find candidate variants from exome data, a sequential strategy was opted for. In the first step, all single nucleotide variants (SNVs) that were not shared among patients were filtered out. The shared variants were further filtered against already reported public databases. For the subtraction of reported SNPs, the data were filtered against dbSNP http://www.ncbi.nlm.nih.gov/projects/SNP/ (accessed on 15 September 2021), The 1000 genomes project http://www.1000genomes.org/ (accessed on 15 September 2021), 6503 samples from the NHLBI Exome Variant Server http://evs.gs.washington.edu/EVS/ (accessed on 15 September 2021), and in the Exome Aggregation Consortium (ExAC) browser http://exac.broadinstitute.org/ (accessed on 15 September 2021), as well as against the in-house exome database of Pakistani samples. Online computational tools SIFT, PolyPhen-2 and Mutation Taster and CADD scores were used to predict the possible impact of mutations on the structure and function of the relevant proteins.

#### In Silico Prediction of Structural Alternation in Mutated Protein

The effect of substituent residue on the structure of the relevant proteins was predicted using the online in silico tool, iTASSER (Iterative Threading ASSEmbly Refinement). iTASSER works on a threading algorithm and models the protein based on crystal structure available in protein databank and template-based fragment assembly simulations. The modeled structure was subjected to Dynamut analysis (available at: http://biosig.unimelb.edu.au/dynamut/ accessed on 29 June 2020) and mutation was induced by replacing ARG at 1169 position with TRP.

### 2.4. Rare Variants Screening through Amplified Fragment Length Polymorphism (AFLP)

In family B, the segregating potential rare variants were screened in a cohort of 508 SCZ patients and fifteen multiplex families of SCZ (51 cases and 47 asymptomatic Controls) from the Pakistani population. A control cohort of 300 unrelated healthy individuals was also screened for all rare variants. The details of primer pairs and restriction enzymes used for each variant are given in Supplementary Table S3.

## 3. Results

### 3.1. CNV Analysis

**Figure 1:**
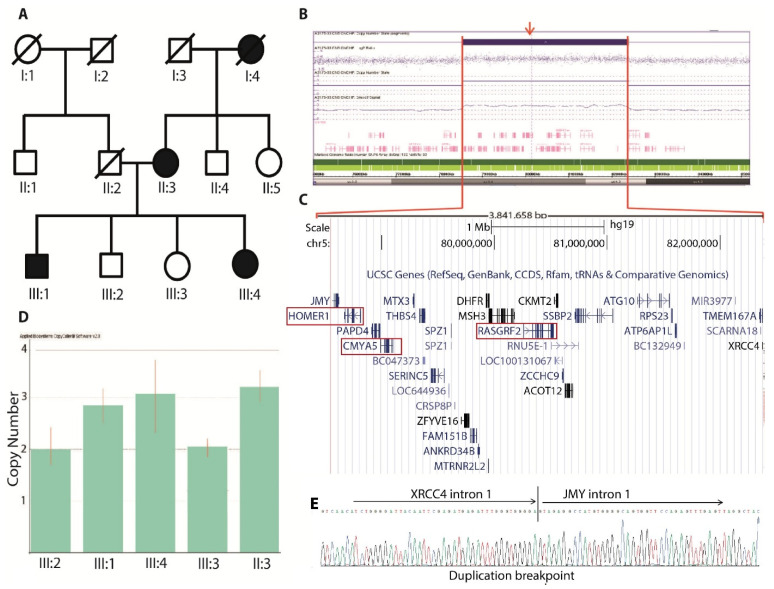
Identification and characterization of duplication at 5q14.1_q14.2 in family A: (**A**) The pedigree of a non-consanguineous Pakistani family with a possible autosomal dominant inheritance of schizophrenia. (**B**) Affymetrix SNP 6.0 data of the proband showing the duplication, ~3.84 Mb in size. (**C**) Screenshot of UCSC genome browser hg19 builds showing 36 reference sequence genes present in the 5q14.1 duplication area. The genes highlighted in red rectangles are previously implicated in schizophrenia. (**D**) Confirmation of the duplication in all available family members using the TaqMan^®^ Copy Number Assay. The predicted copy number state is indicated on the *y*-axis, “C” indicates the control subject. (**E**) The chimeric sequence between the *XRCC4* and *JMY* genes was validated by Sanger sequencing and by a single split mate-pair read.

The genome-wide SNP array analysis of family A revealed 59 CNVs larger than 1 kb in size in index patient III-1 (Supplementary Table S4). We excluded 54 CNVs reported in the Database of Genomic Variants (DGV); the remaining CNVs were filtered to remove those lacking potential candidate genes using PubMed literature mining. Subsequently, four non-DGV reported CNVs were further filtered out due to the functional irrelevance of genes present in these CNVs to neuropsychiatric diseases. Finally, we found a duplication of 3.84 Mb at chromosome 5 (chr5:78553549_82385787dup), spanning 36 genes, which include potential candidate genes for SCZ (Figure 1A–C and Supplementary Table S5).

In family B, none of the detected CNVs were previously reported to be pathogenic, neither did they co-segregate with phenotype. Due to the lack of sufficient evidence of the involvement of CNVs, it was selected for further genomic analyses detailed in Section 3.2.

#### 3.1.1. CNV Confirmation

Real-time TaqMan copy number assay of family A revealed that the 3.84 Mb duplication at 5q14.1_q14.2 is inherited from the affected mother (II-3) (Figure 1D). The affected sister (III-4) is also a carrier of 5q14.1_q14.2 duplication, and while it is absent in the unaffected siblings (III-2, III-3), this confirms the co-segregation of the CNV with the phenotype (Figure 1D). The analysis of 100 SCZ patients from the Pakistani cohort of 508 SCZ patients, described in Section 2.1, using TaqMan copy number assay for the presence of the 5q14.1_q14.2 duplication did not identify any carrier. This, together with its absence in the Database of Genomic Variants (DGV), indicates that it is a very rare variant.

#### 3.1.2. MPS and Breakpoint Detection

In family A, increased coverage of mate-pair sequencing (MPS) reads corresponded exactly with the SNP 6.0 data (Supplementary Figure S1). Moreover, MPS revealed a junction fragment (chr5:82387310-78545653) that indicated a direct tandem duplication (chr5:78545653_82387310) that spans 3.84 Mb, with breakpoints in intron 1 of *JMY* and intron 1 of *XRCC4* (Figure 1E). We did not see any loss or gain at the sequence level in the mate-pair data, indicating that the duplication was not associated with imbalances in breakpoint junctions. The BP junction identified by a single split MPS read was not amplified in healthy siblings or in the healthy controls, confirming that the CNV is present in the patients only (Supplementary Figure S2) The BP junction was further confirmed by PCR amplification and Sanger sequencing (Figure 1E).

In family B, MPS did not reveal any rare imbalanced structural variations hence inversions or translocations were excluded as a possible contribution to the etiology of SCZ in this family.

#### 3.1.3. Breakpoint Screening

To further investigate breakpoint carriers among the Pakistani population, we screened 508 unrelated SCZ patients and 300 unrelated healthy controls through PCR amplification by primers across the breakpoint. The breakpoint was observed neither in these patients nor in the controls. Furthermore, none of the individuals in our cohort of 15 families with SCZ has this duplication; therefore, we concluded that this is a rare CNV identified in SCZ patients in family A.

### 3.2. Whole-Exome Sequencing

**Figure 2:**
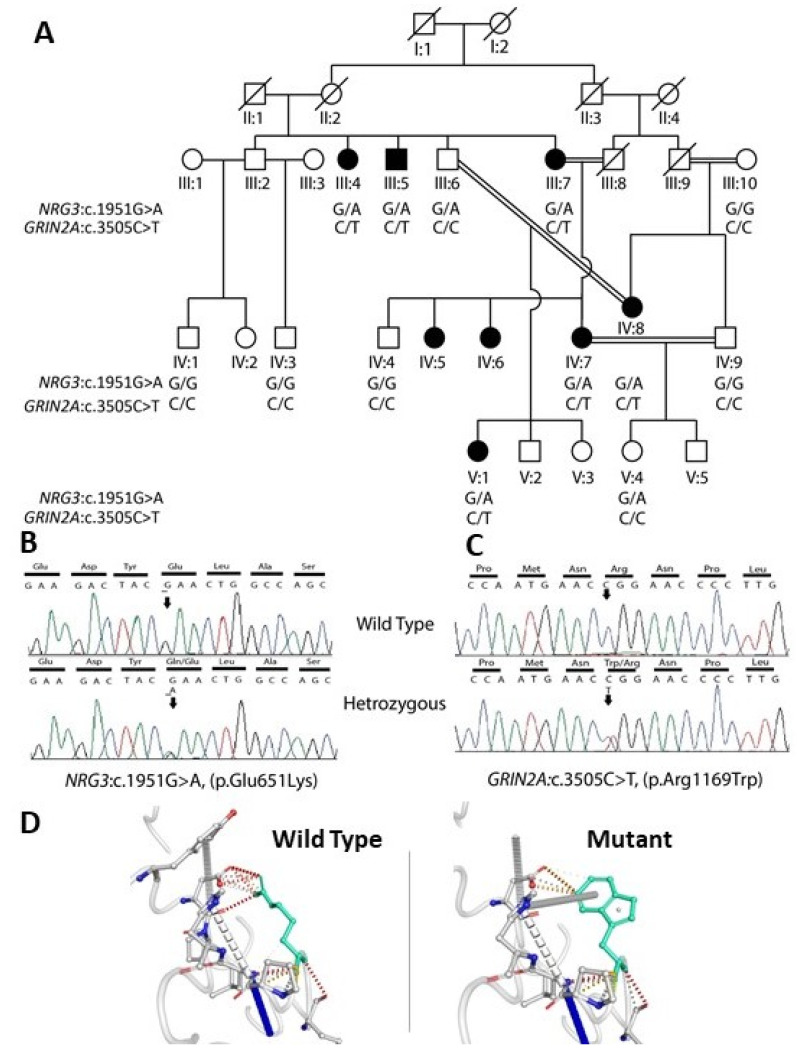
Pedigree and genetic analysis of family B: (**A**) Pedigree of five generations of consanguineous Pakistani family with eight affected individuals (filled symbols), the C/C and G/G represent the wild-type genotype for c.3505C>T and c.1951G>A variants, respectively, which are replaced with C/T and G/A genotype in affected individuals. (**B**) Sequence chromatogram representing *NRG3* variant c. 1951G>A. (**C**) Sequence chromatogram showing part of the *GRIN2A* gene carrying the c.3505C>T variant in wild-type (**top**) and affected individuals (**bottom**). (**D**) iTASSER prediction revealed that the mutation GRIN2A: p.R1169W destabilized the whole structure with increased flexibility of the mutant structure.

For family B, after the exclusion of CNVs and complex structural variations, the hunt for rare variants was focused on the coding portions of the genome. The exome variants shared by three affected individuals were further prioritized based on the functional relevance of the genes. Twelve candidate variants were validated through Sanger sequencing, and, subsequently, segregation analysis was performed. A missense variant ENST00000330684.4: c.3505C>T (NM_001134407.3: p.Arg1169Trp) located in exon 13 of *GRIN2A* gene was co-segregating with disease (Figure 2A,B). Interestingly, in addition to the *GRIN2A* variant, another missense variant ENST00000372141.7: c.1951G>A (NM_001010848.4: p.Glu651Lys) rs138878772 in *NRG3* was also segregated with the phenotype (Figure 2A,B). The remaining variants were not segregating, hence excluded. Both of these variants were predicted by SIFT, Mutation Taster and PolyPhen-2 algorithms to cause a deleterious effect on the protein (Supplementary Table S6). The variant *GRIN2A*: c.3505C>T was also investigated in 15 multiplex families (51 patients and 47 healthy controls), 508 sporadic cases of Pakistani origin and 300 healthy controls through RFLP analysis. We did not find this variant in any of the screened SCZ cases or healthy controls.

### 3.3. In Silico Prediction

The structure was modeled with a −2.05 confidence score and cluster density and structural decoys of 0.0202 and 108, respectively. The cluster density is defined as the number of structure decoys at a unit of space in the SPICKER cluster. A higher cluster density means the structure occurs more often in the simulation trajectory, and therefore, signifies a better quality model.

The prediction outcome revealed that the subject mutation destabilized the whole structure (Figure 2 Bottom panel) with ΔΔG: −0.197 kcal/mol. However, the flexibility of the mutant structure was fairly increased as the value of vibrational and entropy energy value indicate ΔΔSVibENCoM: 0.293 kcal mol^−1^K^−1^.

## 4. Discussion

In this study, we conducted CNV investigations, massively parallel mate-pair sequencing and whole-exome sequencing for two SCZ families of Pakistani origin. The rationale behind this study was that the affected individuals within families would share the same potentially pathogenic variants.

In family A, we identified, and validated to the sequence level, a 3.84 Mb duplication that co-segregated with the phenotype. Among the 36 duplicated genes, *CMYA5*, *HOMER1*, *SERINC5* and *RasGRF2* genes have been repeatedly associated with SCZ and other psychiatric disorders [32,33,34]. However, the involvement of other gene/genes within or around the duplication and effects of conserved regulatory elements cannot be excluded. The functions of each of the potential candidate genes, which are of relevance to SCZ and associated disorders, are explained below.

*HOMER1* is one of the most promising candidate genes within the duplicated (chr5:78545653_82387310) region that is known to be involved in the presynaptic and postsynaptic regulation of glutamate receptor neurotransmission pathway [35]. It encodes for a postsynaptic density (PSD) scaffolding protein that is widely expressed in the central nervous system. It interacts with NMDA receptors and acts as a key regulator of metabotropic glutamate receptors (mGluR1/5) signaling. HOMER1 binds to mGluR1/5 to modulate receptor-mediated downstream Ca^2+^ release [36]. Its dysfunction is reported for loss of structural and functional integrity of glutamatergic neurons in the brains of SCZ patients [37,38]. Homer1 knockout mice are reported to develop behavioral and neurochemical deficits consistent with animal models of SCZ [39]. Homer1 promotes dendritic spine growth, and its loss leads to changes in synaptic proteome [40]. Furthermore, hippocampus overexpression of Homer1a increases the vulnerability to chronic stress and consequently enhances depression-related behavior [41]. Scaffold proteins (i.e., HOMER1, SHANK and GKAP) play crucial roles in PSD and also allow crosstalk among glutamatergic, dopaminergic and serotonergic neurotransmitters [42]. Therefore, any abnormality in PSD scaffold proteins, such as HOMER1, may affect dopamine–glutamate–serotonin interactions [43]. In line with these observations, PDS proteins and mGluRs are the most promising targets for the treatment of psychiatric disorders, especially SCZ [9,44,45,46,47].

Very interestingly, the *HOMER1* gene also houses several circular RNAs (circRNAs) that have been reported to be enriched in synaptic regions, implying their involvement in the regulation of neural development and physiological activity. One of these circHomer1a (Circ0006916, Chr5:78,734,833-78,752,841 GRCh37) has been reported to be down-regulated in the prefrontal cortex and induced pluripotent stem cells derived neurons from SCZ and bipolar disorder patients [48]. Brain region-specific in vivo knockdown of circHomer1a in mouse orbitofrontal cortex has shown that it is capable of regulating the expression of specific isoforms from synaptic plasticity-related genes with relevance for psychiatric disorders [48]. Considering the sensitivity of these genes to the levels of circHomer1a, it is logical to speculate that duplication of this locus can have unfavorable effects on synaptic function and neuronal excitability and thus contribute to the disease.

*RasGRF2* (Ras/Rac guanine nucleotide exchange factor) is another schizophrenia susceptibility gene that expresses heavily in neurons throughout the central nervous system. RasGRF2 activity is known to be essential for NMDA-glutamate receptor-mediated synaptic potentiation and its associated spine enlargement [49]. Moreover, GRF2 is crucial for adult neurogenesis and the survival of neurons [50].

*CMYA5* (Cardiomyopathy-associated protein 5) expresses moderately in the human brain, and it is highly expressed in the heart and skeletal muscle [32]. Several SNPs in *CMYA5*, rs10043986, rs4704591, rs3828611 and rs7714250 have been previously associated with SCZ [31,51,52]. The encoded protein Myospryn is known to interact with various proteins of cytoskeleton, such as α-actinin [53], dystrophin [54], titin [55] and desmin [56], and is thus postulated to mediate neurite outgrowth and axonal transportation in the developing nervous system, the impairment of which can have long-term effects on learning and memory, contributing to the etiology of SCZ [56]. It also interacts with dysbindin, a protein encoded by the DTNBP1 gene, which has been known to be associated with SCZ [57,58]. Dysbindin is an important regulator of glutamate release, and its overexpression leads to the increase in glutamate level by inducing the expression of SNAP25 and synapsin 1 [59]. These together implicate that *CMYA5* duplication might lead to abnormal synaptic plasticity, contributing to the phenotype observed.

The combined Taqman copy number assay and breakpoint screening approaches did not find any more duplication carriers in SCZ patients and controls cohorts. Thus, it appears as a novel, rare and non-recurrent CNV, which segregates with SCZ in family A, providing further evidence that rare CNVs could pose a strong risk for SCZ. It is tempting to speculate that the apparent high burden of SCZ in the present family might be related to an additive effect of the duplication of *CMYA5*, *HOMER1* and *RasGRF2*, with modulating effects on the glutamate neurotransmission pathway, might have contributed to the psychiatric phenotypes of this family. These findings reinforce the possible sensitivity of neuronal plasticity to the doses of these genes and the importance of inherited CNV screening and its role in the pathogenesis of familial SCZ.

In family B, the variant *GRIN2A*: c.3505C>T affects the amino acid 1169 located in the extracellular domain of the protein and is highly conserved among species. *GRIN2A* encodes the GluN2A subunit of N-methyl-D-aspartate (NMDA) receptors, and it is a key mediator of synaptic plasticity throughout the brain. The dysfunction of the *GRIN2A* gene has been previously implicated in the impairment of learning and memory [60], intellectual disability [61], epilepsy [62] and autism, as well as in SCZ [61] and other neurodevelopmental defects [63]. *GRIN2A* is the most strongly associated SCZ gene, emerging from the largest Genome-Wide Association Studies (GWAS) (36,989 cases and 113,075 controls) and is reported as one of the potential therapeutic targets for SCZ because of its involvement in synaptic plasticity and glutamatergic neurotransmission. Recently, WES of 24,248 cases and 97,322 controls revealed ultra-rare coding variants in 10 genes and *GRIN2A* is appeared to confer risk as one of the strongly associated schizophrenia genes [18].

Only a few studies have been reported where exome sequencing identified rare variants with high penetrance segregating in multiplex SCZ families [19,20,27,64,65]. Remarkably, in most of these studies, glutamatergic dysfunction appears to be chiefly involved in SCZ. Protein-altering variants in the NMDA receptor of glutamatergic neurotransmission are involved in multiplex pedigrees SCZ of African American and European American ancestry [19]. Interestingly, another WES of multiplex families highlighted variants involved in the AMPA receptor of the glutamate neurotransmission pathway [20]. NMDA and AMPA glutamate receptors are responsible for calcium-induced potentiation in the brain, and hypofunction in these receptors is the base of glutamatergic abnormalities in SCZ [63,66]. Further supporting evidence to this hypothesis was provided by animal model studies, which showed that the NMDA receptor antagonists lead to reduced hippocampal volume, decreased dendritic spine density and impaired learning and memory, which results in SCZ-like symptoms in non-human primates and rodents [67].

Another missense substitution was also observed in the *NRG3* gene (rs138878772) segregating in this family. *NRG3* belongs to the neuregulin family, and it binds to ERBB4 receptors, inducing neuronal migration and differentiation. Both *NRG3* (encoding neuregulin 3) and its paralog, *NRG1* (encoding neuregulin 1), have been frequently reported as a risk gene for SCZ [68,69,70]. Mutations in interacting partners (NRG1, ERBB4) of NRG3 lead to the hypofunction of glutamatergic neurotransmission. More specifically, mutations in NRG1/ERBB4 lead to impairments in AMPA and NMDA receptors’ spines structure and plasticity [70], and it has been attributed to cognitive deficits observed in SCZ patients [71]. Mice knocked out for *Nrg3*, exhibit behavioral analogs of schizophrenia, such as novelty-induced hyperactivity, impaired prepulse inhibition of the acoustic startle response and deficient fear conditioning [72]. The variant observed in our patients is predicted pathogenic by multiple in silico tools and is likely to disrupt the structure and function of the proteins. The missense variant of *NRG3* is also observed in the heterozygous state in two normal individuals III:3 and V:4. Taken together, it supports the idea that the additive effect of both missense variants (c.3505C>T and c.1951G>A in the *GRIN2A* and *NRG3* gene, respectively) might affect the glutamate neurotransmission that contributed to SCZ in this family.

Taken together, two rare missense variants are found in this SCZ family, suggesting digenic inheritance, which may provide evidence of different gene variants acting synergistically to contribute to the SCZ phenotype. Studying larger families with multiple cases offer a unique opportunity for the identification of genomic variants of large impact and may lead to the identification of novel mechanisms and missing heritability of schizophrenia.

Furthermore, functional studies modeling these variants in cerebral organoids or patient-derived neurons can provide further insights into the pathomechanism.

## 5. Conclusions

In this study, we identified three rare loss of function variants (one structural and two missense) in genes implicated in the glutamate signaling pathway, which are likely to contribute to SCZ pathogenesis in two Pakistani families. Our findings added further evidence to the leading pathophysiological (glutamate) hypothesis of SCZ.

## Data Availability

Data concerning this study are available in the article and Supplementary Materials.

## Supplementary Materials

The following are available online at https://www.mdpi.com/article/10.3390/genes12121899/s1, Table S1: Clinical information of all affected family members of both families, A and B, Table S2: Primer sequences used for break point (bp) characterization. Table S3: Primer sequences used for amplification and Sanger sequencing of exome variants. Table S4: All CNVs present in proband III-1 family A analyzed at 1kb with 10 markers. Table S5: Brief description of all refseq genes present in 5q14.1 duplication, Table S6: Pathogenicity score predicted by in silico tools. Figure S1: Integrative Genomics Viewer (IGV) screenshot of mate-pair data showing the increased coverage of reads corresponding to the duplication. Figure S2: A 385bp polymerase chain reaction (PCR) product spanning the direct tandem duplication junction chr5:82387310:chr5:78545653) is only amplified in duplication carriers.
