# Supplementary material for: Rare Pathogenic Variants in Genes Implicated in Glutamatergic Neurotransmission Pathway Segregate with Schizophrenia in Pakistani Families
Source: Genes (Basel). 2021 Nov 26;12(12):1899. doi: 10.3390/genes12121899 (PMC8700876; doi:10.3390/genes12121899)
Supplement: Supplementary file 1 [file genes-12-01899-s001.zip › genes-1413498-supplementary.pdf]

Supplementary Tables and Figures

Title: Rare Pathogenic Variants in Genes Implicated in Glutamatergic Neurotransmission Pathway Segregate with Schizophrenia in Pakistani Families

Supplementary Table S1: Clinical information of all affected family members of both families A and B

Family ID	Sample ID	Sex (M/F)	Age (Y)	Age of onset (Y)	Education	Marital Status	Hallucinations	Delusions	Disorganized speech	Abnormal psychomotor behavior	Social withdrawal/ lack of emotions	Depres sion	Mania
Family A	III:1	M	39	22	FA	Single	+	+	+	+	-	+	+
Family A	III:4	F	35	24	Matric	Single	+	+	+	+	+	+	+
Family A	II:3	F	67	24	Nil	Married	+	+	+	+	+	+	-
Family B	III:5	M	48	20	Nil	Divorced	+	+	+	+	+	-	+
Family B	IV:8	F	39	20	Primary	Married	+	-	-	-	-	+	-
Family B	III:7	F	63	25	Nil		+	+	-	-	-	-	-
Family B	V:1	F	18	15	Primary	Single	-	+	-	-	+	-	-
Family B	III:4	F	39	21	Primary	Divorced	-	+	-	+	+	+	-
Family B	IV:7	F	36	19	Nil	Married	+	+	+	-	+	-	-

F: Female, M: Male, Y: Years, * +: present, ─: Absent

Supplementary Table S2: Primers sequence used for break point (bp) characterization.

Primer Name	Primer sequence
XRCC4_JMY_dup_F1	TGGCAGGTGAGAGAGAAGAG
XRCC4_JMY_dup_F2	GGTCCTAGGTCAACATCTGG
XRCC4_JMY_dup_R	GGCACTTCACGTACATTCTTTC

Supplementary Table S3: Primer Sequences used for amplification and Sanger sequencing of Exom variatns

Primer Name	Pimer Sequence	Variant	Restriciton Enzyme
GRIN2A-F	TCGCTGGTCTCACTGTGC	c.3505C>T	BSTXI
GRIN2A-R	CCTGAAAACCAAATCAAGCTC		

Supplementary Table S4: All CNVs present in proband III-1 Family A analyzed at 1kb with 10 markers.

CN State	Type	Size (kbp)	Chr	Cytoband	Position	Genes
1.0	LOSS	7,205	1	p13.3	110232961-110240166	*GSTM1*
1.0	LOSS	7,258	1	p31.1	72756100-72763358	-
4.0	GAIN	42,651	1	p31.1	72768485-72811136	-
1.0	LOSS	51,04	1	p36.11	25595934-25646974	*RHD*
1.0	LOSS	36,868	1	p36.33	61722-98590	*OR4F5*
4.0	GAIN	30,828	1	q21.3	152555754-152586582	*LCE3C, LCE3B*
0.0	LOSS	5,597	1	q25.1	174796543- 174802140	*RABGAP1L*
1.0	LOSS	10,256	2	p23.3	24601459- 24611715	-
1.0	LOSS	53,186	2	q11.2	98108991- 98162177	*ANKRD36B*
1.0	LOSS	8,136	2	q31.2	180413349- 180421485	*ZNF385B*
1.0	LOSS	112,537	3	q26.1	162513446- 162625983	-
3.0	GAIN	5,002	3	q29	192877889- 192882891	-
1.0	LOSS	24,858	4	p16.1	9461217- 9486075	-
1.0	LOSS	2,716	4	p16.1	10228770- 10231486	-
1.0	LOSS	11,006	4	q12	55104742- 55115748	*PDGFRA*
1.0	LOSS	164,678	4	q13.2	69375335-69375335	*UGT2B17, UGT2B15*
1.0	LOSS	7,267	4	q22.1	91287151-91287151	*FAM190A*
1.0	LOSS	10,682	4	q32.3	168616719-168616719	-
4.0	GAIN	3.05	4	q34.1	172374871-172374871	-
3.0	GAIN	3,832,238	5	q14.1	78553549-78553549	*JMY, HOMER1, PAPD4, CMYA5, MTX3, THBS4, SERINC5, LOC644936, SPZ1, CRSP8P, ZFYVE16, FAM151B, ANKRD34B, DHFR, MTRNR2L2, MSH3, RASGRF2, RNU5E, RNU5D, CKMT2, LOC100131067, ZCCHC9, ACOT12, SSBP2, ATG10, RPS23, ATP6AP1L, TMEM167A, SCARNA18, XRCC4*
1.0	LOSS	4,707	6	p21.31	33937986- 33937986	-
1.0	LOSS	42,684	6	p21.32	32454510-32497194	*HLA-DRB5*
4.0	GAIN	2,727	6	p22.3	16517338-16520065	*ATXN1*
1.0	LOSS	5,346	6	q14.1	81284176-81289522	-
4.0	GAIN	15,431	6	q22.33	129556516-129571947	*LAMA2*
1.0	LOSS	1,183	6	q26	161032088-161033271	*LPA*
1.0	LOSS	7,016	7	q21.3	97395447-97402463	-
1.0	LOSS	9,327	7	q34	142476706-142486033	*TRY6, PRSS2*
4.0	GAIN	115,953	7	q35	143911611-144027564	*OR2A42, OR2A1, OR2A9P, OR2A20P, OR2A7, LOC728377, CTAGE4*
1.0	LOSS	9,903	8	p21.2	24974430-24984333	-
4.0	GAIN	8,619	8	p23.2	2247646-2256265	-
1.0	LOSS	6,825	8	q23.3	115634894-115641719	-
1.0	LOSS	1,178,324	9	p11.2	46038840-47217164	*KGFLP1*
0.0	LOSS	10.37	9	p21.3	23363102-23373472	-
4.0	GAIN	1,139	9	q21.11	72028457-72029596	-
4.0	GAIN	2,792	10	p12.2	24375250-24378042	*KIAA1217*
3.0	GAIN	1,213,224	10	q11.22	46966534-48179758	*SYT15, GPRIN2, PPYR1, LOC643650, LOC728643, ANXA8, ANXA8L1, FAM25B, FAM25C, FAM25G, AGAP9, LOC642826, FAM35B2, ANTXRL, ANXA8L2, FAM21B, CTSLL2*
4.0	GAIN	5,901	11	p15.4	4968116-4974017	*OR51A4*
1.0	LOSS	7,572	12	p11.21	33299790-33307362	-
1.0	LOSS	4,991	12	q24.13	112894499-112899490	*PTPN11*
4.0	GAIN	31,078	14	q32.33	106530351-106561429	-
1.0	LOSS	11,614	15	q11.1	20569938-20581552	-
1.0	LOSS	204,831	15	q11.2	22383188-22588019	*OR4N4, OR4N3P, REREP3*
1.0	LOSS	296,827	16	p13.12	14780641-15077468	*PLA2G10, ABCC6P2, NOMO1, MIR3179-2, MIR3179-1, MIR3179-3, MIR3180-3, MIR3180-1, MIR3180-2, NPIP, PDXDC1*
1.0	LOSS	17,122	17	q21.2	39413397-39430519	-
1.0	LOSS	16,288	17	q21.31	41259367-41275655	*BRCA1*
3.0	GAIN	323.62	17	q21.31	44428668-44752288	*ARL17A, ARL17B, NSFP1, LRRC37A2, NSF*
4.0	GAIN	4,592	18	q12.3	38260785-38265377	-
4.0	GAIN	2,317	18	q22.1	64959194-64961511	-
1.0	LOSS	38,233	20	p13	1560909-1599142	*SIRPB1*
4.0	GAIN	12,958	21	q22.3	45486269-45499227	*TRAPPC10*
1.0	LOSS	43,302	22	q11.23	24353500-24396802	*LOC391322, GSTT1, GSTTP2*

2.0	GAIN	28,661	X	p11.23	48968008-48996669	*GPKOW*
2.0	GAIN	85,371	X	q21.31	88599251-88684622	-
2.0	GAIN	85,105	X	q21.31	89849760-89934865	-
2.0	GAIN	51,615	X	q21.31	91162117-91213732	*PCDH11X*
2.0	GAIN	14,274	X	q21.32	92331809-92346083	-
2.0	GAIN	22,756	X	q26.3	134857306-134880062	*CT45A2, CT45A4*
2.0	GAIN	28,393	X	q27.3	143402115-143430508	-

Supplementary Table S5: Brief description of all refseq genes present in 5q14.1 duplication

Gene	protein	Function	Disease Association	References
JMY	Junction-Mediating And -Regulatory Protein	Acts as a nuclear p53/TP53-cofactor and a cytoplasmic regulator of actin dynamics.	*hypoxia, breast cancer*.	Coutts *et al*: 2011
HOMER1	Scaffolding protein	Binds and cross-links cytoplasmic regions of GRM1, GRM5, ITPR1, DNM3, RYR1, RYR2, SHANK1 and SHANK3 due to its role in PSD it is a most convincing candidate gene.	neurological diseases, Schizophrenia	Luo *et al*: 2012
PAPD4	PAP-Associated Domain-Containing Protein 4	PAPD4 is involved in a mechanism that controls miRNA-related activities in the nervous system.	sleeping sickness	Kinjoa *et al*: 2013
CMYA5	Cardiomyopathy Associated 5	Serve as an anchoring protein that mediates the subcellular compartmentation of protein kinase A (PKA).	Schizophrenia, skeletal muscle regeneration	Chen *et al*: 2010
MTX3	Metaxin 3	Function in transport of proteins into the mitochondrion.	charcot-marie-tooth neuropathy x, system lymphoma	
THBS4	Thrombospondin 4	Adhesive glycoprotein that mediates cell-to-cell and cell-to-matrix interactions	breast cancer, mycetoma,	
SERINC5	Serine Incorporator 51	Carrier protein involve in lipid biosynthesis.	Borderline personality (BP) disorder	Lubke *et al*: 2013
LOC644936	Actin, Beta Pseudogene	-	-	-
SPZ1	Spermatogenic Leucine Zipper 11	Involved in mitogen-activate protein kinase (MAPK) signaling pathway.	-	-
CRSP8P	Mediator Complex Subunit 27 Pseudogene	-	-	-
ZFYVE16	Zinc Finger, FYVE Domain Containing 16	Belong to zinc finger family of proteins, implicated in regulating membrane trafficking in the endosomal pathway.		Seet *et al*: 2004
FAM151B	Family With Sequence Similarity 151, Member B	Protein coding gene.		
ANKRD34B	Ankyrin Repeat Domain 34B	Ankrd34b might be a positive regulator of neurogenesis and a negative regulator of adipogenesis.		Doss *et al*: 2010
DHFR	Dihydrofolate Reductase	Converts dihydrofolate into tetrahydrofolate, a methyl group shuttle required for the de novo synthesis of purines, thymidylic acid, and certain amino acids.	Isosporiasis, megaloblastic anemia	Milic *et al*: 2012
MTRNR2L2	MT-RNR2-Like 2	Plays a role as a neuroprotective and antiapoptotic factor.	Alzheimer's disease, neuronitis.	
MSH3	MutS Homolog 3	Involve in nucleotide mismatch and double-strand break repair.	lynch syndrome, mucoepidermoid carcinoma	Kumar *et al*: 2013
RASGRF2	Ras Protein-Specific Guanine Nucleotide-Releasing Factor 2	Involve in synaptic plasticity also regulates alcohol-induced reinforcement by dopamine release.	orofacial cleft, and colon cancer	Stacey *et al*: 2012
RNU5E	RNA, U5E small nuclear 1	RNU5E-1 is RNA gene from snRNA class with largely unknown functions.	-	-
RNU5D	RNA, U5D Small Nuclear 1	RNU5D-1 is an RNA gene and is affiliated with the snRNA class.	-	-
CKMT2	Basic-Type Mitochondrial Creatine Kinase	CKMT2 is essential to energy metabolisms, responsible for the transfer of high energy phosphate from mitochondria to the cytosolic carrier, creatine.	prostate rhabdomyosarcoma, and myopathy	
LOC100131067	non-coding RNA			
ZCCHC9	Zinc Finger, CCHC Domain Containing 9	May play roles in the Mitogen-Activated Protein Kinase (MAPK) signaling transduction pathway.	intrahepatic cholangiocarcinoma, and cholangiocarcinoma	Zhou *et al*: 2008
ACOT12	Acyl-CoA Thioesterase 12	Hydrolyze the thioester bond of acetyl-CoA in the cytosol in the liver.	pericarditis, and cleft palate	Horibata *et al*: 2013
SSBP2	Single-Stranded DNA Binding Protein 2	Involved in the maintenance of genome stability.	Acute lymphocytic leukemia and lissencephaly.	Huang *et al*: 2009
ATG10	Autophagy Related 10	Play a role in autophagy.	Myelodysplastic syndromes, and several types of cancers	Flanagan *et al*: 2012

RPS23	Ribosomal Protein S23	A typical for genes encoding ribosomal proteins.	pasteurellosis, and ascariasis,	
ATP6AP1L	ATPase, H+ Transporting, Lysosomal Accessory Protein 1-Like	Proton-transporting ATP synthase activity.		
TMEM167A	Transmembrane Protein 167A	Is affiliated with the lncRNA class.		
SCARNA18	Small Cajal Body-Specific RNA 18	Noncoding RNAs involved in RNA processing.		
XRCC4	X-Ray Repair Cross-Complementing Protein 4	Involved in DNA non-homologous end joining (NHEJ) required for double-strand break repair and V(D)J recombination	xeroderma pigmentosum, group d, and artemis deficiency,	Brandi *et al*: 2013

Supplementary Table S6: Pathogenicity score predicted by *in silico* tools

**Prediction tool**	**Function**	**Prediction**	
		***NRG3*:NM_001010848.4: c.1951G>A; p.(Glu651Lys)**	***GRIN2A*:NM_001134407.3: c.3505C>T; p.(Arg1169Trp)**
**PROVEAN**	Protein variation effect analyzer	Neutral (-0.483)	Neutral (-1.357)
**CADD**	Combined Annotation Dependent Depletion	27.2	22.8
**SNPs & GO**	Variant effect prediction using gene ontology terms	Disease causing	Disease causing
**PANTHER**	Protein analysis through evolutionary Relationships	Probably damaging	Probably damaging
**SIFT**	Predict effects of nonsynonymous / missense variants	Neutral 0.190	Disease 0.020
**SNAP**	Prediction of nonsynonymous functional effects	Disase 0.685	Disease 0.610
**Meta-SNP**	Meta-predictor of disease causing variants	Diseases 0.593	Disease 0.659

Supplementary Figure S1: Integrative Genomics Viewer (IGV) screenshot of mate-pair data showing the increased coverage of reads corresponding to the duplication

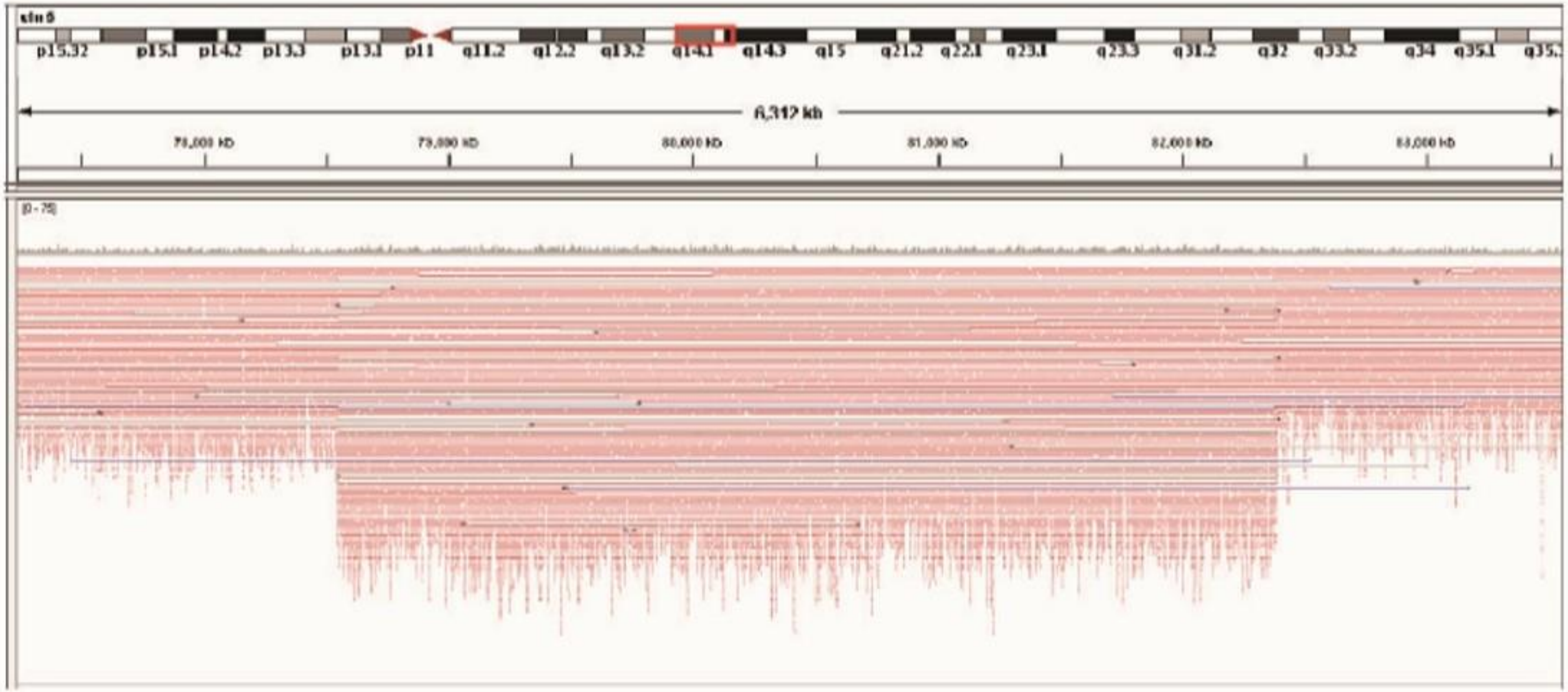

Supplementary Figure S2: A 385bp polymerase chain reaction (PCR) product spanning the direct tandem duplication junction chr5:82387310:chr5:78545653) is only amplified in duplication carriers

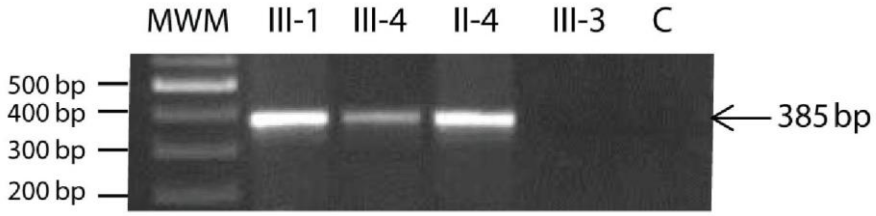

Supplementary references

1. Coutts AS, Pires IM, Weston L *et al:* Hypoxia-driven cell motility reflects the interplay between JMY and HIF-1α. *Oncogene* 2011; **30**: 4835–4842.
2. Luo P, Li X, Fei Z, Poon W: Scaffold protein Homer 1: Implications for neurological diseases. *Neurochem Int* 2012; **61**: 731–738.
3. Kinjo ER, Higa GS, de Sousa E *et al:* A possible new mechanism for the control of miRNA expression in neurons. *Exp Neurol* 2013; **248**: 546–558.
4. Chen X, Lee G, Maher BS *et al:* GWA study data mining and independent replication identify cardiomyopathy-associated 5 (CMYA5) as a risk gene for schizophrenia. *Mol Psychiatry* 2011; 16: 1117–1129.
5. Lubke GH, Laurin C, Amin N *et al:* Genome-wide analyses of borderline personality features. *Mol Psychiatry* 2013; 1-7.
6. Seet LF, Liu N, Hanson BJ *et al:* Endofin recruits TOM1 to endosomes. *J. Biol. Chem* 2004; **279**: 4670-4679.
7. Doss MX, Wagh V, Schulz H, Kull M: Global transcriptomic analysis of murine embryonic stem cell-derived brachyury+ (T) cells. *Genes Cells* 2010; 209–228.
8. Milic V, Jekic B, Lukovic L *et al:* Association of dihydrofolate reductase (DHFR) -317AA genotype with poor response to methotrexate in patients with rheumatoid arthritis. *Clin Exp Rheumatol* 2012; **30**:178-183.
9. Kumar C, Williams GM, Havens B, Dinicola MK, Surtees JA: Distinct requirements within the Msh3 nucleotide binding pocket for mismatch and double-strand break repair. *J Mol Biol* 2013; **425**: 1881-98.
10. Stacey D, Bilbao A, Maroteaux M *et al:* RASGRF2 regulates alcohol-induced reinforcement by influencing mesolimbic dopamine neuron activity and dopamine release. Proc *Natl Acad Sci U S A* 2012; **109**: 21128-21133.
11. Zhou A, Zhou J, Yang L *et al:* A nuclear localized protein ZCCHC9 is expressed in cerebral cortex and suppresses the MAPK signal pathway. *J Genet Genomics* 2008; **35**: 467–472.
12. Horibata Y, Ando H, Itoh M, Sugimoto H: Enzymatic and transcriptional regulation of the cytoplasmic acetyl-CoA hydrolase ACOT12. *J Lipid Res* 2013; **54**: 2049-2059.
13. Huang J, Gong Z, Ghosal G, Chen J: SOSS complexes participate in the maintenance of genomic stability. *Mol Cell* 2009; **35**: 384-393.
14. Flanagan MD, Whitehall SK, Morgan BA: Structural insights into Atg10-mediated formation of the autophagy-essential Atg12-Atg5 conjugate. *Structure* 2012; **20**:1244-1254.
15. Mahaney BL, Hammel M, Meek K, Tainer JA, Lees-Miller SP: XRCC4 and XLF form long helical protein filaments suitable for DNA end protection and alignment to facilitate DNA double strand break repair. *Biochem Cell Biol* 2013; **91**: 31-41.
